# Diminution of Voltage Threshold Plays a Key Role in Determining Recruitment of Oculomotor Nucleus Motoneurons during Postnatal Development

**DOI:** 10.1371/journal.pone.0028748

**Published:** 2011-12-09

**Authors:** Livia Carrascal, Jose Luis Nieto-González, Blas Torres, Pedro Nunez-Abades

**Affiliations:** 1 Departamento de Fisiología, Universidad de Sevilla, Sevilla, Spain; 2 Departamento de Fisiología Médica y Biofísica, Universidad de Sevilla, Sevilla, Spain; University of Houston, United States of America

## Abstract

The size principle dictates the orderly recruitment of motoneurons (Mns). This principle assumes that Mns of different sizes have a similar voltage threshold, cell size being the crucial property in determining neuronal recruitment. Thus, smaller neurons have higher membrane resistance and require a lower depolarizing current to reach spike threshold. However, the cell size contribution to recruitment in Mns during postnatal development remains unknown. To investigate this subject, rat oculomotor nucleus Mns were intracellularly labeled and their electrophysiological properties recorded in a brain slice preparation. Mns were divided into 2 age groups: neonatal (1–7 postnatal days, n = 14) and adult (20–30 postnatal days, n = 10). The increase in size of Mns led to a decrease in input resistance with a strong linear relationship in both age groups. A well-fitted inverse correlation was also found between input resistance and rheobase in both age groups. However, input resistance versus rheobase did not correlate when data from neonatal and adult Mns were combined in a single group. This lack of correlation is due to the fact that decrease in input resistance of developing Mns did not lead to an increase in rheobase. Indeed, a diminution in rheobase was found, and it was accompanied by an unexpected decrease in voltage threshold. Additionally, the decrease in rheobase co-varied with decrease in voltage threshold in developing Mns. These data support that the size principle governs the recruitment order in neonatal Mns and is maintained in adult Mns of the oculomotor nucleus; but during postnatal development the crucial property in determining recruitment order in these Mns was not the modifications of cell size-input resistance but of voltage threshold.

## Introduction

The control of posture and movement requires an understanding of how the activation of motor unit is organized. Henneman and colleagues published seminal papers in the sixties that provide a detailed account of motor unit properties, motoneuron recruitment properties, and how the relation between these two sets of properties could be summarized in terms of a unifying principle that they called the “size principle” [1]–[3]. The size principle describes the sequence of recruitment of motor units in producing movements with increased force or speed: the first motoneurons (Mns) to be recruited are those that have slower conduction velocity and that connect with muscle fibers that contract with lesser force, slower contraction time, and lower susceptibility to fatigue [1], [2], [4], [5]. Classically, the order of recruitment of Mns has been considered to depend on the input resistance, such that small Mns with less surface area would have higher input resistance and would produce a larger voltage drop for a given synaptic input than larger ones. Therefore, small Mns would also reach voltage threshold at a lower level of synaptic input. The size principle assumes that Mns with different sizes have a similar voltage threshold and that the functional synaptic inputs tend to be equalized among Mns [1], [2]. A Mn property that might broaden the range of intrinsic excitability is the persistent inward current that is activated by much less depolarization in the most excitable Mns than in less excitable ones [6], [7]. Synaptic inputs to Mns are also known to expand or compress the recruitment threshold range or to reverse the usual order in which Mns are recruited [8]–[13]. In spinal Mns of the zebrafish, the recruitment threshold was not dictated by the input resistance but was instead determined by a combination of specific biophysical properties and the strength of the synaptic currents [14].

The combination of intrinsic properties that might establish the hierarchy in Mns excitability (cell size, voltage threshold, and/or membrane resistivity) remains essentially unknown during postnatal development. In developing hypoglossal Mns a decrease in input resistance during the first two weeks after birth has been reported, together with a later cell size growth during the third postnatal week [15], [16]. In other words, decrease in input resistance did not parallel with increase in cell size during maturation in this pool of Mns. Therefore, a correlation between anatomical and passive membrane properties was not found [17] and input resistance diminution was likely attributable to a proliferation of leak potassium channels leading to a decrease in specific membrane resistance [18], [19]. These data put into question the presence of the size principle in newborn Mns.

The anatomical and electrophysiological features of the oculomotor nucleus Mns have been separately analysed during postnatal development [20]–[22]. A rapid growth in cell surface took place between 1 and 10 days old; and then dendrites, but not the soma, continued with gradual growth up to postnatal day 30. During postnatal development major changes in complexity and length of dendrites have also been reported [22]. Furthermore, the resting membrane potential did not significantly change with age, while input resistance and time constant diminished drastically within a short time after birth (1–5 postnatal days). Therefore, the temporal course of the maturation in cell size is longer than that found in input resistance. The rheobase (current intensity required to reach spike threshold) declined with postnatal development; this result is surprising since it means that growth in cell size did not lead to an increase in rheobase, which obviously challenges the size principle during postnatal development. This work was addressed to examine if the recruitment of the Mns according to the size principle is already present in newborn Mns, and how the modification of active membrane properties with development may challenge the size principle. With this aim, we studied the relationships between cell size, input resistance, rheobase and depolarization voltage in developing Mns of the oculomotor nucleus. To accurately accomplish these goals, the present study combined, in the same Mn, intracellular labeling with electrophysiological recording in an in vitro brain slice preparation.

## Materials and Methods

Experiments were carried out from 1 to 7-day-old (P1–P7) and from 20 to 30-day-old (P20-P30) Wistar rats (5–140 g) of both sexes. These age groups were selected because early after birth they show an increase in cell size and a decrease in input resistance, while physiological and anatomical features of oculomotor nucleus Mns remain essentially unchanged after P20 [21], [22]. This study was carried out in strict accordance with the recommendations in the Guide for the Care and Use of Laboratory Animals of the European Community Directive 2003/65 and the Spanish Royal Act 120/2005. The protocol was approved by the Committee on the Ethics of Animal Experiments of the University of Seville (Permit Number: 0724-2009). Rats were anesthetized with sodium pentobarbital (50 mg/Kg) and quickly decapitated. The method for obtaining the slices (400 µm of thickness) is fully detailed in a prior study [21], [22]. Slices including the oculomotor nucleus were first incubated in a chamber containing cold sucrose–ACSF for 35–45 min, and then transferred to a second chamber containing ACSF at a temperature of 32±1°C. Individual slices were finally transferred to the recording chamber and perfused at 2 mL/min (MPII, Harvard Apparatus, Holliston, MA, USA) with ACSF bubbled with 95% O2–5% CO2 (pH 7.4; 32±1° C). The composition of ACSF was as follows (data are in mM): 126 NaCl, 2 KCl, 1.25 Na2HPO4, 26 NaHCO3, 10 glucose, 2 MgSO4, and 2 CaCl2. For sucrose/ACSF solution, the 126 NaCl was substituted with 240 mM sucrose.

All recorded neurons were identified as Mns by their antidromic activation from the root of the third nerve and by the collision test [21]. The micropipettes used for recordings were filled with a 3 M KCl (50–90 MΩ) solution containing 2% neurobiotin (Vector Laboratories, Burlingame, CA). The electrophysiological properties of the Mns were first recorded, and then labeled intracellularly with neurobiotin. Resting membrane potentials were measured as the difference between the intracellular and extracellular potentials after withdrawing the recording electrode from the cell. Input resistance was determined by passing a series of negative current pulses (500 ms, 1 Hz) with 0.1 nA increments through the microelectrode. Input resistance was calculated as the slope of the voltage – current plot. The rheobase was the minimum current injected (50 ms, 1 Hz) that generated action potentials in 50% of the cases. The depolarization voltage was the increase in membrane potential required to bring the cell to the spike threshold. To determine the spike threshold, the action potential recording was differentiated; spike onset was the value of the membrane potential at which the first derivative surpassed 10 V/s. Voltage threshold was calculated by adding depolarization voltage to the resting membrane potential. Neurobiotin was iontophoresed into the Mns by passing a depolarizing current pulse (0.2–1 nA) during 500 msec at 1 Hz. The duration of iontophoresis ranged from 15 to 60 minutes. After intracellular injection of neurobiotin, the slices were immersed in fixative (4% paraformaldehyde) at 4°C for 1–2 days, and then moved to 30% sucrose in phosphate buffer (0.05 M; pH 7.4) at 4°C overnight. An avidin-biotin-peroxidase complex (Vectastain Elite; Vector Laboratories) was used to reveal neurobiotin tracer (detailed in Carrascal et al., 2009). For morphological analysis, Mns were first reconstructed in 3D using the Neurolucida system (MicroBrightField, Williston, VT) on an Olympus microscope (BX61; Olympus, Tokyo, Japan) at 63 magnification. The somal perimeter was outlined to measure the somal membrane area. The dendrites were reconstructed by taking multiple measurements (approximately every 5–10 µm), assigning X, Y, Z, and diameter values. Morphometric measurements were extracted from the reconstruction files in Neuroexplorer software (MicroBrightField). The slice shrinkage was measured on the X, Y, and Z axes and corrections were applied for each neuron [22].

**Figure 1:**
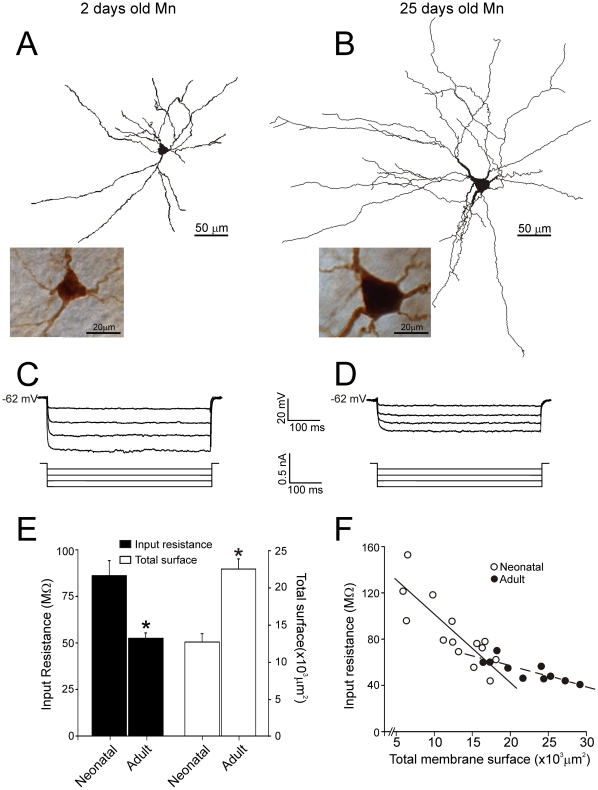
Ce ll-size and input resistance in neonatal and adult Mns within oculomotor nucleus. **A-B.** Reconstructions of representative neonatal **(A)** and adult **(B)** Mns. Photomicrographs illustrate the somata and main dendrite trees. **C-D.** Voltage membrane responses to negative current pulses evoked in cells illustrated in A,B. **E.** Histograms illustrating mean input resistance and total membrane surface area in neonatal and adult Mns. **F.** Relationship between the total membrane surface and the input resistance for neonatal (open circles) and adult (solid circles) Mns. Lines are best fit to the raw data. Asterisks indicate statistical significance.

**Figure 2:**
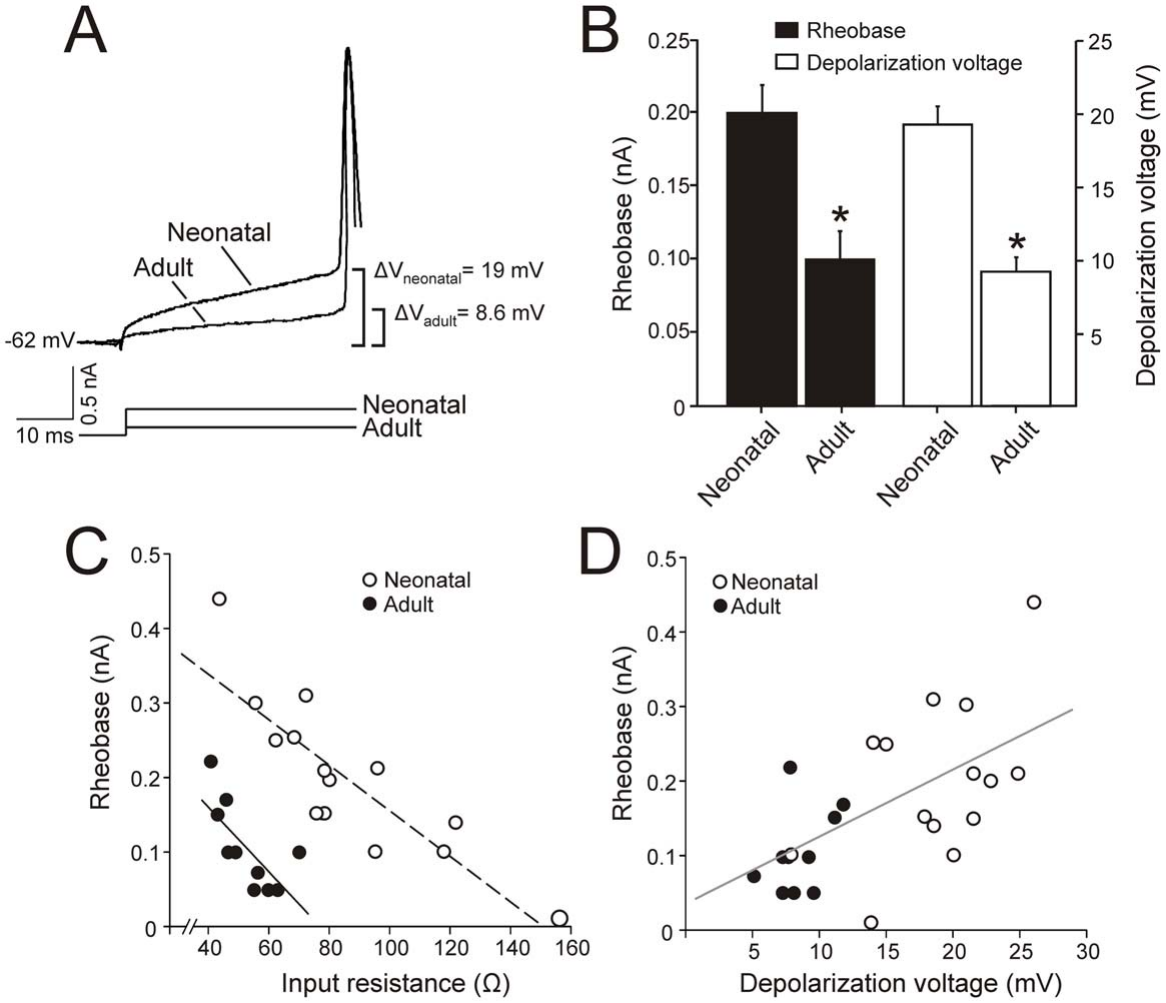
Spike recruitment in neonatal and adult Mns as a function of input resistance and depolarization voltage. A. Minimum current required (rheobase) to evoke an action potential in the representative cases illustrated in Figure 1A,B. The values of depolarization voltage are also noted (ΔV). **B**. Histograms illustrating mean rheobase and depolarization voltage in neonatal and adult Mns. **C.** Relationship between input resistance and rheobase for neonatal (open circles) and adult (solid black circles) Mns. Lines are best fit to the raw data. **D.** Relationship between depolarization voltage and rheobase for neonatal and adult Mns. The gray line is best fit to the raw data. Asterisks indicate statistical significance.

All Mns included in the analysis showed a stable resting membrane potential of at least −55 mV or below, an action potential larger than 60 mV, and fired repetitively in response to depolarization pulses of one second. Several parameters were analyzed: resting membrane potential, input resistance, rheobase, depolarization voltage and voltage threshold. Reconstructed Mns had a healthy appearance, with no evidence of impalement damage to the somal membrane, minimal extracellular dye leakage, and a good contrast with low background labeling of the tissue. The dendritic arborization was contained within the slices, because terminations corresponded to natural endings (final diameter ranged between 0.2 and 1 µm) as opposed to cut branches. Among the 55 Mns recorded and injected with neurobiotin, only 31 showed good labeling and 24 of these well-stained Mns were fully reconstructed. These Mns were grouped in neonatal (P1–P7, n = 14) and young adult rats (P20-P30, n = 10). Data for analysis were plotted and quantified in Origin software 7.5 (Originlab Corporation, Northampton, MA). All statistical analyses were carried out on the raw data, significant differences between neonatal and adult age groups were determine by using a one-way ANOVA test. The significance level was established at *P*<0.05. Finally, the best fit to the raw data was calculated, represented as a dashed line (for P1-P7) and a continuous line (for P20-P30) in Figures 1 and 2.

## Results

Membrane surface area of the neonatal and adult oculomotor nucleus Mns showed differences in size of cell bodies and dendrites. Neonatal Mns had a smaller cell body and displayed shorter dendrites than adult Mns (see the representative examples in figure 1A,B). Total membrane surface of neonatal Mns (n = 14) ranged from 5,886 to 18,107 µm^2^ (12,626±1,129 µm^2^, mean ± standard error), while in adult Mns (n = 10) it ranged from 16,413 to 29,154 µm^2^ (22,433±1,359 µm^2^). Differences in the membrane surface between Mns of both age groups were significant (Fig. 1E). Other anatomical characteristics were similar in neonatal and adult Mns: the cell bodies showed a polygonal shape, the number of primary dendrites was between four and eight, dendrites were observed emerging from different edges of the somata and spreading radially in all directions, these dendrites were mainly confined within oculomotor nucleus boundaries, and exhibited similar dendritic complexity (measured as the number of branch segments and terminal endings). Additionally, all recorded Mns showed a stable membrane potential which was not significantly different between neonatal (−61.3±2.3 mV) an adult (−62.1±3.4 mV) Mns. These Mns were silent at rest and required depolarizing current injection to elicit an action potential. Injections of negative current pulses evoked membrane potential hyperpolarizations. Figure 1C-D shows that the neonatal Mn exhibited larger hyperpolarizations than those found in the adult Mn in response to current pulses of the same intensities. Input resistance ranged from 43.9 to 159.9 MΩ (86.1±8.1 MΩ) in neonatal Mns, while it ranged from 40.8 to 70.1 MΩ (52.5±2.9 MΩ) in adult Mns. The diminution in input resistance with postnatal age was statistically significant (Fig. 1E). Therefore, cell size increased and input resistance decreased with postnatal development, and the ranges of values for membrane surface area and input resistance for neonates (3-fold and 4-fold, respectively) were considerable greater than those for the more mature Mns (2-fold in both cases).

To evaluate the size principle in oculomotor nucleus Mns, we first studied whether input resistance co-varies with motoneuron membrane surface (Fig. 1F). A significant well-fitted linear relationship was found between these two parameters in the adult group of Mns (r = −0.83; P<0.002). We also checked if these two parameters co-varied after birth and found a significant well-fitted linear relationship between membrane surface and input resistance (r = −0.82; P<0.001) in neonatal Mns. In other words, input resistance was dependent on size in both adult and neonatal Mns and as expected the largest Mns had the smallest input resistance irrespective of age. Nevertheless, it should be noted that slopes of the relationship between size and input resistance were different in neonatal and adult age groups. Furthermore, the slope that represents this relationship was steeper for the neonatal Mns (58.5 MΩ/10,000 µm^2^) than for the adult one (17.6 MΩ/10,000 µm^2^). The differences in slopes coursed in parallel to the differences in ranges of values for membrane surface area and input resistance between neonates and adult Mns. Finally, when size and input resistance of neonatal and adult Mns were combined in a single group and the linear relationship between these parameters was quantified, a significant negative co-variation was also found (r = -0.8; P<0.01 not illustrated).

The depolarizing current (rheobase) and the voltage increment (depolarization voltage) required to evoke an action potential is shown in figure 2A for representative neonatal and adult Mns. These recording were obtained in the same Mns illustrated in figure 1A–B, and these cells had a resting membrane potential of −62 mV. For these representative examples, the values of rheobase (0.2 nA *vs.* 0.1 nA), depolarization voltage (19 mV *vs.* 8.6 mV), and voltage threshold (-43 mV *vs.* −53.4 mV) were lower in the adult Mn than in the neonatal one. Rheobase values ranged from 0.01 to 0.44 nA in Mns of the first postnatal week, and from 0.05 to 0.22 nA in adult Mns. The depolarization voltage ranged from 7.8 to 24.8 mV for the neonatal age group, and from 5.1 to 14.4 mV for the adult age group. The voltage threshold ranged from −55 to -38.7 mV in neonatal Mns and from -56.1 mV to -46.8 mV in adult Mns. A decrement in rheobase from 0.20±0.02 to 0.10±0.01 nA, in depolarization voltage from 18.9±1.2 to 9.2±0.8 mV and in voltage threshold from −42.7±2.1 mV to −52.7±1.4 mV occurred with maturation. The differences in these parameters were statistically significant when neonatal and adult age groups were compared (Fig 2B).

Since cell size and input resistance showed a well fitted linear relationship, the second major point in evaluating the size principle in oculomotor nucleus Mns was to study whether rheobase co-varies with input resistance. These two parameters were significantly well-fitted to a linear relationship in both neonatal (r = −0.85; P<0.001) and adult (r = -0.7; P< 0.02) Mns (Fig. 2C). These data indicate that smaller Mns had higher input resistance and lower rheobase. Therefore, the sequence of recruitment was determined by cell size – input resistance in neonatal Mns and maintained in adult Mns. In addition, if the recruitment of the Mns obeys the size principle throughout postnatal development, a well-fitted co-variation between rheobase and input resistance would be expected when data from adult and neonatal Mns were combined in a single group. However, rheobase did not co-vary with input resistance when data were grouped. In other words, assuming an “ohmic” response of the membrane, which is exclusively grounded on input resistance, the expected values of rheobase in adult Mns should be higher than those found (Fig. 2C). In consequence, the increase in cell size of the Mns with postnatal age led to a decrease in input resistance but *did not* produce an increase in rheobase. Indeed, the comparison of neonatal (n = 6) and adult (n = 4) Mns with similar sizes (between 15,000 and 20,000 µm^2^), dendritic complexity and input resistant, showed different values of rheobase and voltage threshold (Table 1). As observable in the table, rheobase was lower than 0.1 nA in adult Mns and higher than 0.1 nA in neonatal ones; voltage threshold was more negative than −50 mV in adult Mns and less negative than −50 mV in neonatal ones. Therefore, the size principle guides recruitment in neonatal and adult oculomotor nucleus Mns when studied separately, but cell size-input resistance is not the only biophysical property that determines rheobase during development.

**Table 1:** Contribution of voltage threshold to spike recruitment in Mns during postnatal development.

Postnatal age(days)	Surface (µm^2^)	Terminals	RMP (mV)	Rin(MΩ)	Rheobase(nA)	Depolarization voltage (mV)	Voltagethreshold (mV)
4	16,307	26	−61	72.5	0.31	18.6	−42
5	16,658	24	−65	78	0.21	24.8	−40
6	18,107	28	−64	62.3	0.25	16	−48
7	15,599	34	−63	76.2	0.15	18	−45
7	17,355	29	−62	43.9	0.44	24	−36
7	15,202	31	−62	55.6	0.3	21	−41
**Neonatal**	16.538±442.1	28.6±1.5	−62.8±0.6	64.8±5.5	0.27±0.04	20.4±1.4	−42±1.7
22	17,319	38	−63	59.6	0.05	7.4	−56
25	18,235	32	−60	70.1	0.1	7.6	−52
26	16,413	23	−61	60	0.05	9.7	−51
27	19,899	29	−62	49.2	0.1	7.4	−55
**Adult**	17966±743.9	30.5±3.1	−61.5±0.6	59.0±4.90	0.07±0.01	8.0±0.5	−53.5±1.6

Neonatal and adult oculomotor nucleus motoneurons (Mns) exhibiting similar membrane surface, dendritic complexity

(number of terminals) and input resistance (Rin) showed lower values in rheobase, depolarization voltage and voltage threshold

in adult Mns. Values in neonatal and adult files are the mean ± standard error.

Previous findings demonstrate that Mns with similar cell size and input resistance have different rheobase when neonatal and adult Mns are compared. According to Ohm's law (rheobase  =  depolarization voltage/input resistance), rheobase differences should be attributed to changes in depolarization voltage with development. To test this proposal, rheobase was plotted as a function of the depolarization voltage for each age group and no co-variation was obtained either in neonatal or in adult epochs (Fig 2.D). That means, for a given value of depolarization voltage different rheobase were found irrespective of age. However, when these data were combined in a single group, rheobase co-varied with depolarization voltage (r = 0.64; P<0.001). Indeed, the values of depolarization voltage were higher in neonatal Mns than in adult ones, and the distribution found in adult Mns was narrower and shifted towards lower values. According to these results, depolarization voltage and, therefore, voltage threshold has a strong influence in determining the sequence of recruitment of oculomotor nucleus Mns during development.

## Discussion

his work investigates the size principle in developing Mns from the rat oculomotor nucleus, studying how the somatodendritic shaping impacts input resistance and rheobase. Neonatal and adult Mns showed a strong linear relationship between size and input resistance, and between input resistance and rheobase. These relationships support that the size principle is established early after birth and is kept in the adult Mns. Nevertheless, the most noticeable finding reported here is that voltage threshold (an active membrane property that was not linked to cell size), and not input resistance, could play a key role in determining the recruitment order of Mns during postnatal development.

### Cell size, input resistant and postnatal development

The size principle determines the orderly recruitment of motor units in a wide variety of muscles [3], [23]–[26]. In spinal Mns, the recruitment order is establish predominantly by intrinsic membrane properties like size, current threshold and input resistance [11]. Mn size determines input resistance in adults; for instance, small Mns in abducens nucleus have high input resistance [27]. Present data demonstrate a significant well-fitted linear regression between total membrane surface and input resistance, and between input resistance and rheobase in adult oculomotor nucleus Mns. Therefore, the recruitment order in oculomotor nucleus adult Mns is plausibly dictated by the size principle. In addition, the same functional relationships were found in this work in neonatal Mns, leading to the notion that the size principle also governs recruitment order of Mns early after birth. However, it should be noted that the slope of the relationships between size and input resistance was three times higher in neonatal Mns (i.e., for a given amount of membrane growth it has a lower impact on input resistance in adult Mns than in neonatal ones). The relationship between cell size and input resistance has been studied in detail in phrenic and genioglossal Mns during postnatal development. In these populations of Mns, there were postnatal periods in which a large drop of input resistance was found, but the total surface area did not change, and it was proposed that the installation of leak potassium channels may lead to the input resistance reduction [16], [18], [19], [25], [28]. Additionally, in spinal Mns from studies in wild type *versus* mutant rats, it has been suggested that morphology contributed only partially to the modification in input resistance [29]. Alternatively, the wide ranges of values for membrane surface area and input resistance in newborn Mns could indicate that individual Mns grow at different rate.

All in all, it can be suggested that cell size determines input resistance, but its contribution should be tempered depending on the postnatal age.

### Size principle, voltage threshold and postnatal development

In Mns from the oculomotor nucleus a significant well-fitted linear relationship was observed between the size and rheobase in both neonatal and adult Mns, which demonstrates that the size principle is established early after birth and it is maintained in the adult. However, size and rheobase did not correlate when the results from neonatal and adult Mns were combined in a single group. Furthermore, increase in size in these Mns with maturation did not lead to an increase in rheobase during the postnatal development. Rather, cell growth in developing Mns was accompanied by a decrease in rheobase. This finding could be due to the fact that input resistance decline with postnatal age did not produce an increase in rheobase, as reported in other pools of Mns [15]. A premise inherent to the size principle is that Mns with different sizes have similar voltage threshold; the lack of correlation between input resistance and rheobase indicates that this premise does not apply to postnatal development in oculomotor nucleus Mns.

Present results shows that the depolarization voltage needed to reach spike threshold is lower in adult Mns than in neonatal Mns, which means a decrease in spike voltage threshold with development. Therefore, it is plausible that this change could lead to a decrease in the rheobase with postnatal age in these Mns. Thus, the excitability of these Mns increases with maturation despite the decrease of the membrane resistance. The diminution in the voltage threshold of oculomotor nucleus Mns with age could be due to an increase in persistent Na^+^ conductance and long-lasting Ca^2+^ currents. Such inward currents, activated at the subthreshold level, produce excitation and may be involved in Mn recruitment [6], [7], [30]–[32]. These conductances have been reported in brainstem Mns [7], [33]–[38]. If these currents are modulated during postnatal maturation, and they influence spike threshold, they might explain the lower depolarization voltage in adult oculomotor nucleus Mns. Other possible factors could act concurrently to diminish voltage threshold with postnatal age; for instance, recent findings have demonstrated plasticity in the axonal initial segment that influences cell excitability [39]. It has been proposed that the location and extent of this spike triggering zone can be modified by synaptic deprivation or chronic depolarization [40]–[42]. Kuba and colleagues [42] have reported that synaptic deprivation causes increase of the axon initial segment length, which is accompanied by greater voltage gated sodium current, and a diminution in both current and voltage threshold to trigger action potential. Further studies of the modifications in ionic currents (persistent inward current, voltage gated sodium current…) and in the axon initial segment with postnatal development should provide further insights to better understand the electrophysiological bases of the decrease in rheobase and voltage threshold.

### Recruitment in developing Mns in the context of the maturation of the oculomotor system

Early after birth (up to postnatal day 10) the circuitry transmitting the signal from vestibular nuclei to the ocular Mns is mostly established [43]–[45]; Mns grow and lower their input resistance [20]–[22]; rodent extraocular muscles are very immature [46]; their muscle fibers contain supernumerary motor nerve terminals [47]; and extraocular muscle twitching occurs [48]. Later, by postnatal day 11, and at all ages thereafter postsynaptic membranes of muscle fibers appear fully covered by single nerve terminals [47], eyelids open about postnatal day 12 and eye movements evoked by visual and vestibular stimuli occur from about three weeks after birth [49], a time in which Mns exhibit adult firing properties [21], [22], [50]–[52]. It is widely accepted that synaptic circuitry, Mn excitability and extraocular muscles mature jointly. The patterns of oculomotor Mn-specific activity may be determinant for muscle fiber phenotype, as reported in spinal Mns [53]; motor units are refined during postnatal development strengthening some synapse and eliminating others [47]; muscle-derived factors (neurotrophins) are essential for neuronal survival during development and their range of actions support the phasic and tonic activities of Mns in conducting eye movement [54]; lastly, synaptic inputs to Mns play an important role in determining recruitment threshold [52], [55]. With postnatal development, more active Mns have competitive advantages in muscle synaptic refinement [47], [56]. In this work we have demonstrated that Mns of different age groups, showing similar sizes, somato-dendritic morphologies, and input resistances, exhibited distinct voltage and recruitment threshold. The decrease in voltage threshold with development increases Mn excitability, and this physiological change contributes to some extent to the refinement of the motor unit.

## References

[1] Henneman E, Somjen G, Carpenter DO (1965). Functional significance of cell size in spinal motoneurons.. J Neurophysiol.

[2] Mendell LM (2005). The size principle: a rule describing the recruitment of motoneurons.. J Neurophysiol.

[3] Duchateau J, Enoka RM (2011). Human motor unit recordings: origins and insight into the integrated motor system.. Brain Res.

[4] Bawa P, Binder MD, Ruenzel P, Henneman E (1984). Recruitment order of motoneurons in stretch reflexes is highly correlated with their axonal conduction velocity.. J Neurophysiol.

[5] Cope TC, Clark BD (1991). Motor-unit recruitment in the decerebrate cat: several unit properties are equally good predictors of order.. J Neurophysiol.

[6] Lee RH, Heckman CJ (1988). Bistability in spinal motoneurons in vivo: systematic variations in persistent inward currents.. J Neurophysiol.

[7] Powers RK, Nardelli P, Cope TC (2008). Estimation of the contribution of intrinsic currents to motoneuron firing based on paired motoneuron discharge records in the decerebrate cat.. J Neurophysiol.

[8] Davies L, Wiegner AW, Young RR (1993). Variation in firing order of human soleus motoneurons during voluntary and reflex activation.. Brain Res.

[9] Heckman CJ, Binder MD (1993). Computer simulations of the effects of different synaptic input systems on motor unit recruitment.. J Neurophysiol.

[10] Powers RK, Robinson FR, Konodi MA, Binder MD (1993). Distribution of rubrospinal synaptic input to cat triceps surae motoneurons.. J Neurophysiol.

[11] Haftel VK, Prather JF, Heckman CJ, Cope TC (2001). Recruitment of cat motoneurons in the absence of homonymous afferent feedback.. J Neurophysiol.

[12] Binder MD, Heckman CJ, Powers RK (2002). Relative strengths and distributions of different sources of synaptic input to the motoneurone pool: implications for motor unit recruitment.. Adv Exp Med Biol.

[13] Kang Y, Saito M, Toyoda H, Sato H (2010). Recruitment of masseter motoneurons by the presumed spindle Ia inputs.. Prog Brain Res.

[14] Gabriel JP, Ausborn J, Ampatzis K, Mahmood R, Eklöf-Ljunggren E (2011). Principles governing recruitment of motoneurons during swimming in zebrafish.. Nat Neurosci.

[15] Nunez-Abades PA, Spielmann JM, Barrionuevo G, Cameron WE (1993). In vitro electrophysiology of developing genioglossal motoneurons in the rat.. J Neurophysiol.

[16] Nunez-Abades PA, Cameron WE (1995). Morphology of developing rat genioglossal motoneurons studied in vitro: relative changes in diameter and surface area of somata and dendrites.. J Comp Neurol.

[17] Nunez-Abades PA, Cameron WE (1997). Relationship between membrane properties and cell size of developing rat genioglossal motoneurons studied in vitro.. Neurosci Lett.

[18] Cameron WE, Nunez-Abades PA (2000). Physiological changes accompanying anatomical remodeling of mammalian motoneurons during postnatal development.. Brain Res Bull.

[19] Cameron WE, Nunez-Abades PA, Kerman IA, Hodgson TM (2000). Role of potassium conductances in determining input resistance of developing brain stem motoneurons.. J Neurophysiol.

[20] Carrascal L, Nieto-Gonzalez JL, Cameron WE, Torres B, Nunez-Abades PA (2005). Changes during the postnatal development in physiological and anatomical characteristics of rat motoneurons studied in vitro.. Brain Res Brain Res Rev.

[21] Carrascal L, Nieto-Gonzalez JL, Nunez-Abades P, Torres B (2006). Temporal sequence of changes in electrophysiological properties of oculomotor motoneurons during postnatal development.. Neuroscience.

[22] Carrascal L, Nieto-Gonzalez JL, Torres B, Nunez-Abades P (2009). Changes in somatodendritic morphometry of rat oculomotor nucleus motoneurons during postnatal development.. J Comp Neurol.

[23] Fleshman JW, Munson JB, Sypert GW, Friedman WA (1981). Rheobase, input resistance, and motor-unit type in medial gastrocnemius motoneurons in the cat.. J Neurophysiol.

[24] Gustafsson B, Pinter MJ (1984). Relations among passive electrical properties of lumbar alpha-motoneurones of the cat.. J Physiol.

[25] Cameron WE, Jodkowski JS, Fang H, Guthrie RD (1991). Electrophysiological properties of developing phrenic motoneurons in the cat.. J Neurophysiol.

[26] Mantilla CB, Seve YB, Zhan WZ, Sieck GC (2011). Diaphragm Motor Unit Recruitment in Rats.. Respir Physiol Neurobiol.

[27] Grantyn R, Grantyn A (1978). Morphological and electrophysiological properties of cat abducens motoneurons.. Exp Brain Res.

[28] Nunez-Abades PA, Pattillo JM, Hodgson TM, Cameron WE (2000). Role of synaptic inputs in determining input resistance of developing brain stem motoneurons.. J Neurophysiol.

[29] Elbasiouny SM, Amendola J, Durand J, Heckman CJ (2010). Evidence from computer simulations for alterations in the membrane biophysical properties and dendritic processing of synaptic inputs in mutant superoxide dismutase-1 motoneurons.. J Neurosci.

[30] Russo RE, Hounsgaard J (1999). Dynamics of intrinsic electrophysiological properties in spinal cord neurones.. Prog Biophys Mol Biol.

[31] Hornby TG, McDonagh JC, Reinking RM, Stuart DG (2002). Effects of excitatory modulation on intrinsic properties of turtle motoneurons.. J Neurophysiol.

[32] Gabrielaitis M, Buisas R, Guzulaitis R, Svirskis G, Alaburda A (2011). Persistent sodium current decreases transient gain in turtle motoneurons.. Brain Res.

[33] Gueritaud JP (1988). Electrical activity of rat ocular motoneurons recorded in vitro.. Neuroscience.

[34] Mosfeldt Laursen A, Rekling JC (1989). Electrophysiological properties of hypoglossal motoneurons of guinea-pigs studied in vitro.. Neuroscience.

[35] Viana F, Bayliss DA, Berger AJ (1993). Calcium conductances and their role in the firing behavior of neonatal rat hypoglossal motoneurons.. J Neurophysiol.

[36] Berger AJ, Bayliss DA, Viana F (1996). Development of hypoglossal motoneurons.. J Appl Physiol.

[37] Powers RK, Binder MD (2003). Persistent sodium and calcium currents in rat hypoglossal motoneurons.. J Neurophysiol.

[38] Li Y, Gorassini MA, Bennett DJ (2004). Role of persistent sodium and calcium currents in motoneuron firing and spasticity in chronic spinal rats.. J Neurophysiol.

[39] Ogawa Y, Rasband MN (2008). The functional organization and assembly of the axon initial segment.. Curr Opin Neurobiol.

[40] Grubb MS, Burrone J (2010). Activity-dependent relocation of the axon initial segment fine-tunes neuronal excitability.. Nature.

[41] Gründemann J, Häusser M (2010). Neuroscience: A plastic axonal hotspot.. Nature.

[42] Kuba H, Oichi Y, Ohmori H (2010). Presynaptic activity regulates Na(+) channel distribution at the axon initial segment.. Nature.

[43] Curthoys IS (1979). The vestibulo-ocular reflex in newborn rats.. Acta Otolaryngol.

[44] Curthoys IS (1981). The organization of the horizontal semicircular duct, ampulla and utricle in the rat and guinea pig.. Acta Otolaryngol.

[45] Rüsch A, Lysakowski A, Eatock RA (1998). Postnatal development of type I and type II hair cells in the mouse utricle: acquisition of voltage-gated conductances and differentiated morphology.. J Neurosci.

[46] Spencer RF, Porter JD (2006). Biological organization of the extraocular muscles.. Prog Brain Res.

[47] Fox MA, Tapia JC, Kasthuri N, Lichtman JW (2011). Delayed synapse elimination in mouse levator palpebrae superioris muscle.. J Comp Neurol.

[48] Seelke AM, Karlsson KA, Gall AJ, Blumberg MS (2005). Extraocular muscle activity, rapid eye movements and the development of active and quiet sleep.. Eur J Neurosci.

[49] Faulstich BM, Onori KA, du Lac S (2004). Comparison of plasticity and development of mouse optokinetic and vestibulo-ocular reflexes suggests differential gain control mechanisms.. Vision Res.

[50] Tsuzuki S, Yoshida S, Yamamoto T, Oka H (1995). Developmental changes in the electrophysiological properties of neonatal rat oculomotor neurons studied in vitro.. Neurosci Res.

[51] Nieto-Gonzalez JL, Carrascal L, Nunez-Abades P, Torres B (2007). Phasic and tonic firing properties in rat oculomotor nucleus motoneurons, studied in vitro.. Eur J Neurosci.

[52] Carrascal L, Luque MA, Sobrino V, Torres B, Nunez-Abades P (2010). Postnatal development enhances the effects of cholinergic inputs on recruitment threshold and firing rate of rat oculomotor nucleus motoneurons.. Neuroscience.

[53] Buller AJ, Eccles JC, Eccles RM (1960). Interactions between motoneurones and muscles in respect of the characteristic speeds of their responses.. J Physiol.

[54] Davis-López de Carrizosa MA, Morado-Díaz CJ, Tena JJ, Benítez-Temiño B, Pecero ML et al (2009). Complementary actions of BDNF and neurotrophin-3 on the firing patterns and synaptic composition of motoneurons.. J Neurosci.

[55] Nieto-Gonzalez JL, Carrascal L, Nunez-Abades P, Torres B (2009). Muscarinic modulation of recruitment threshold and firing rate in rat oculomotor nucleus motoneurons.. J Neurophysiol.

[56] Barber MJ, Lichtman JW (1999). Activity-driven synapse elimination leads paradoxically to domination by inactive neurons.. J Neurosci.

